# An Unusual Presentation of Cholesterol Embolization Syndrome

**DOI:** 10.7759/cureus.16993

**Published:** 2021-08-08

**Authors:** Basel Abdelazeem, Sarah Ayad, Abdul-Fatawu Osman, Rudin Gjeka, Arvind Kunadi

**Affiliations:** 1 Internal Medicine, McLaren Health Care/Flint/MSU, Flint, USA; 2 Internal Medicine, Rutgers-New Jersey Medical School/Trinitas Regional Medical Center, Elizabeth, USA; 3 Internal Medicine, Michigan State University, East Lansing, USA; 4 Cardiology, McLaren Regional Medical Center, Flint, USA; 5 Internal Medicine/Nephrology, McLaren Health Care/Flint/MSU, Flint, USA

**Keywords:** case report, cholesterol embolization syndrome, atheroembolism, hypertension, renal failure

## Abstract

Cholesterol embolization syndrome (CES) is a rare presentation of systemic atherosclerosis, which commonly presents in patients with risk factors of coronary artery disease and usually occurs after cardiac or vascular procedures. Laboratory tests are nonspecific, and imaging studies may visualize the plaque. Management includes supportive care directed to relieve the end-organ damage. The prognosis of CES is poor, with high mortality of up to 29% if the CES resulted in atheroembolic renal disease (AERD). In our report, we present a 90-year-old Caucasian female who was diagnosed with CES and complicated with AERD. The patient did not undergo any cardiac or vascular procedures. This case highlights the importance of considering CES and AERD as a potential cause of renal failure, especially in high-risk patients, even if the patients did not have any history of cardiac or vascular intervention.

## Introduction

Cholesterol embolization syndrome is a rare presentation of systemic atherosclerosis. It is more common in older men with risk factors of coronary artery disease like hypertension, hypercholesterolemia, and smoking. Cholesterol embolization syndrome (CES) refers to embolize the cholesterol crystal or atheroma plaques to different body organs [1]. CES occurs commonly after invasive arterial procedures, including cardiac catheterization, carotid endarterectomy or stenting, cardiac, aortic, and vascular surgery [1-2]. Saklayen et al. reported a 2% incidence rate of atheroembolic renal failure after coronary angiography [3]. Cholesterol crystal embolization rarely occurs spontaneously. Herein, we report such a case and highlight the importance of considering a CES in patients with known atherosclerotic diseases presented with acute renal failure and did not have any previous invasive arterial procedures.

## Case presentation

A 90-year-old Caucasian female with a past medical history of hypertension and chronic kidney disease (CKD) stage 3 and dementia was presented to the emergency department (ED) due to the lethargy of one week. The patient complained of a severe headache -- abrupt in onset, constant, and associated with nausea and vomiting. The patient denied fever, sinus pressure, chest pain, shortness of breath, neck pain, photophobia, blurred vision, loss of consciousness, or seizure activity. According to the daughter, the patient did not take her home medication for the last two days. Home medication included hydralazine, clonidine, and metoprolol.

The patient was found to have hypertension emergency with a blood pressure of 200/107 mmHg, heart rate of 88 beats per minute (bpm), a temperature of 98.5°F, and saturating 97% on room air. The patient was awake but not alert or oriented. Pupils were equal and reacted to light. Extraocular movements were intact. On auscultation, S1 and S2 were regular. No murmur, rubs, gallops, or carotid bruits. Normal bilateral equal breathing sounds without wheezes, rhonchi, or rales. Peripheral pulse felt bilateral. No pedal edema was noted. The patient also had intact motor, sensory, and cranial nerves examination. The abdomen is soft, symmetric, and non-tender without distention excepted for mild bilateral flank tenderness on palpation. Bowel sounds are present and normoactive in all four quadrants. No masses, hepatomegaly, or splenomegaly are noted. 

The patient was started on nicardipine drip; subsequently, the blood pressure was better controlled at 150/70 mmHg. Labs were summarized in Table 1. Urinalysis was negative, and the hepatitis panel was negative.

**Table 1:** Laboratory workup. WBC, white blood cell; BUN, blood urea nitrogen; GFR, glomerular filtration rate; IgG, immunoglobulin G

Labs	Value	Reference
WBC	7.3	4.5-11 10*9/L
Eosinophils	1	0-6%
Hemoglobin	13	12-17 g/dL
Platelets	163	140-440 10*9/L
BUN	53	8-28 mg/dL
Creatine	2.1	0.4-1 mg/dL
Estimated GFR	22	90-120 mL/min/1.73 m2
Troponin	0.00	0.00-0.09 ng/mL
Erythrocyte sedimentation rate	52	2-15 mm/h
Serum cholesterol level	148	142-200 mg/dL
Anti-neutrophil cytoplasmic antibody, IgG	<1:20	<1:20
C4 Complement	29	10-40 mg/dL
C3 Complement	120	90-180 mg/dL

**Figure 1:**
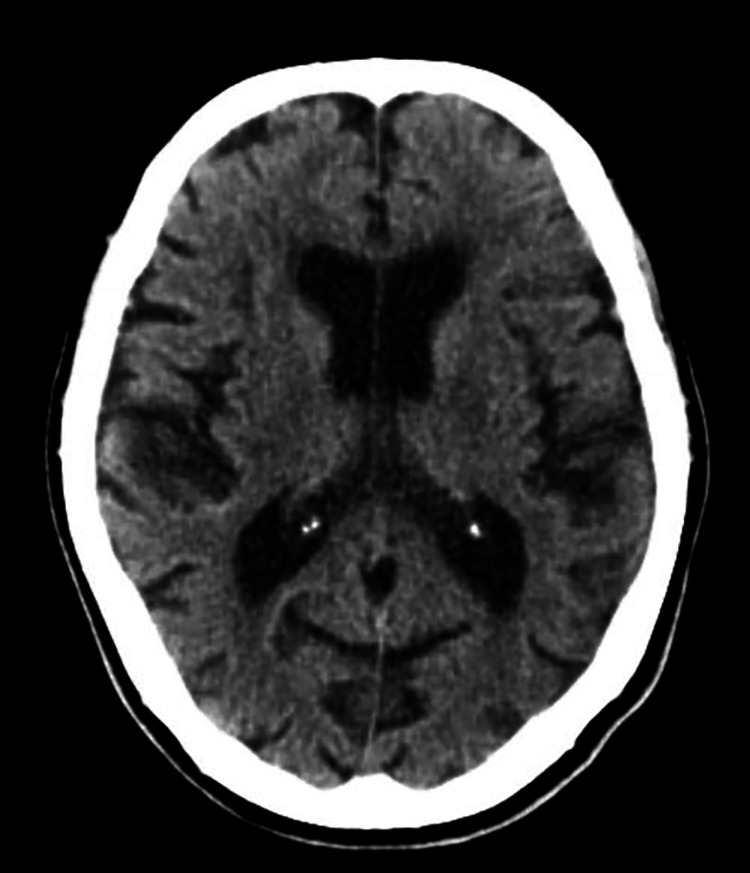
CT head without contrast. CT head without contrast showed moderate diffuse atrophy and no acute intracerebral changes, hemorrhage, or infarcts.

CT head without contrast showed moderate diffuse atrophy and no acute intracerebral changes, no hemorrhage, or infarcts (Figure 1). The echocardiogram showed an estimated left ventricular ejection fraction of 70%-75% and normal global left ventricular size, wall thickness with no apparent regional wall abnormalities, and no plaques visualized in the thoracic aorta. Nicardipine drip was weaned off, and the blood pressure was controlled with oral metoprolol. The patient was discharged home with an outpatient follow-up.

**Figure 2:**
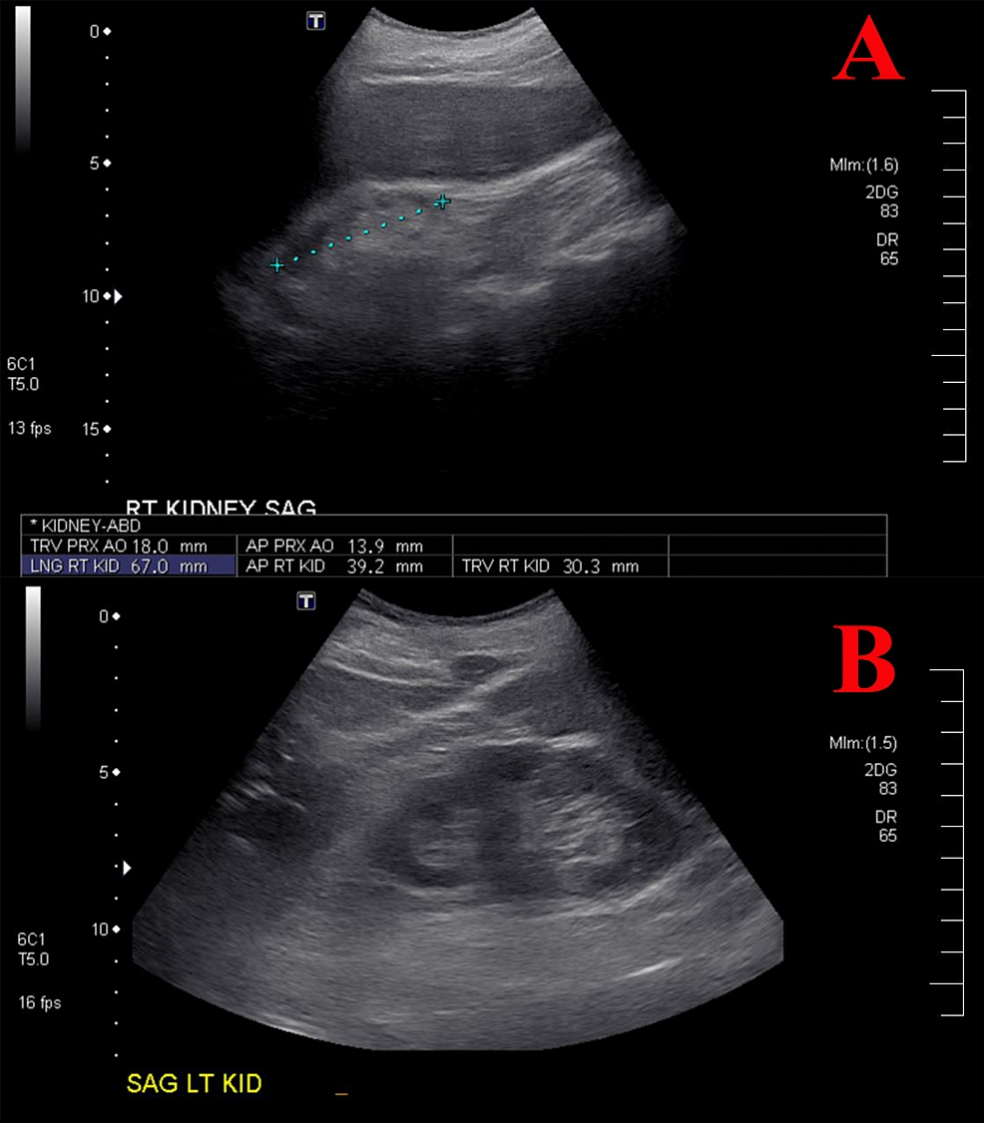
Kidney ultrasound. A: right kidney was severely atrophic (70.3 mm x 39.2 mm x 30.3 mm) with increased echo density without hydronephrosis. B: left kidney was measuring (103.7 mm x 43.9 mm x 39.3 mm) and demonstrated generalized cortical thinning without hydronephrosis.

**Figure 3:**
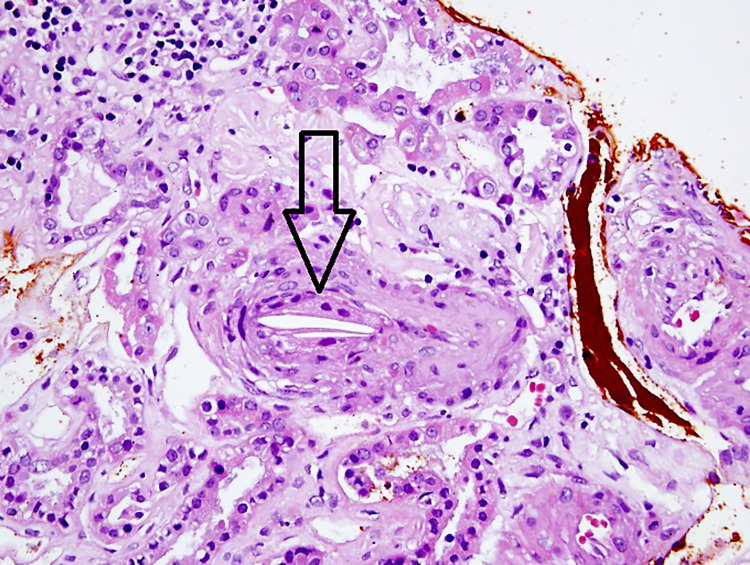
Histopathology. Hematoxylin and eosin stain (40x) revealed a small artery with atheroembolus (cholesterol embolus) with an adjacent cellular reaction. Arrow pointed to cholesterol cleft.

During the follow-up period, the patient developed acute kidney injury on top of CKD. A kidney ultrasound showed the left kidney measured (103.7 mm x 43.9 mm x 39.3 mm) and demonstrated generalized cortical thinning without hydronephrosis. The right kidney was severely atrophic (70.3 mm x 39.2 mm x 30.3 mm) with increased echo density without hydronephrosis (Figure 2). In the setting of acute renal failure and atrophic right kidney, renal biopsy was obtained from the normal left kidney. The specimen consisted of cores of tan tissue with a width of 0.1 cm and a length of 0.5-2 cm. Histopathology revealed a small artery with atheroembolus (cholesterol embolus or cholesterol cleft) with adjacent cellular reaction (Figure 3). The patient was started on a high-intensity statin (atorvastatin) 80 mg daily to treat CES. But, the patient had a worsening kidney function over three days durations, and she was started on dialysis on the fifth day. A few months later, the patient had a non-ST segment elevated myocardial infarction. The patient and her family declined any surgical intervention and decided to proceed with hospice care, and the patient passed away later.

## Discussion

Atheroembolic renal disease occurs when CES is affecting the kidneys, and up to 50% of CES cases are complicated with atheroembolic renal disease (AERD) [1-2]. Haas et al. investigated the causes of acute renal insufficiency in 259 patients aged 60 years or older who underwent a renal biopsy as part of their work-up. Haas et al. reported cholesterol embolization in 18 biopsies (7.1% of all cases) and only three patients with prebiopsy clinical suspicion of CES [4]. Our patient also did not have any clinical suspicion of CES and underwent renal biopsy, which revealed CES as a case of her worsening kidney function. 

Atheroembolic renal disease can be acute within seven days of the inciting event, subacute arises within weeks or months, or chronic occurs within years. It is caused by the occlusion of small arteries of the kidney, especially the arcuate and interlobar renal arteries leading to secondary ischemic atrophy with progressive decline in renal function [5]. In contrast to AERD, thromboembolism is usually presented in patients with atrial fibrillation or cardiac arrhythmia. Thromboembolism leading to complete occlusion of the renal arterial system causes renal infarction [6].

The CES also affects the brain, skin, gastrointestinal tract and can lead to systemic manifestation. Table 2 summarizes the symptoms and signs of CES on different organs other than the kidney.

**Table 2:** Clinical manifestations of CES on different body organs. CES, cholesterol embolization syndrome; TIA, transient ischemic attack

Systemic manifestation	Brain	Skin	Gastrointestinal manifestation
Fever, fatigue, weight loss, anorexia, and myalgia	TIA, stroke, memory loss, confusion, headache, and Hollenhorst plaques	Livedo reticularis, gangrene, cyanosis (Blue toe syndrome), ulceration, nodules, and purpura	Ischemic colitis, pseudopolyp formation, necrotizing pancreatitis, acalculous necrotizing cholecystitis

Laboratory tests are nonspecific and may reveal elevated inflammatory markers, including erythrocyte sedimentation rate, C-reactive protein, elevated white blood cell count with hypereosinophilia, anemia, and thrombocytopenia [2]. Imaging may visualize the plaque. Transcranial doppler may be helpful to detect cerebral microemboli. Echocardiography, CT, or MRI can detect prominent atherosclerotic plaques in the aorta or major branches [7]. Definitive diagnosis of CES was made only by biopsy in the presence of cholesterol cleft within the arterioles. Our patient's laboratory tests were nonspecific for CES, and she underwent an echocardiogram but did not detect any plaque in the thoracic aorta. Due to an acute decline in the patient's kidney function, renal ultrasound was obtained and revealed kidney atrophy which warranted further investigation with biopsy that revealed cholesterol cleft.

There is no definitive medical management for AERD and only supportive care can be provided including diuresis and renal replacement therapy. Surgical or endovascular removal or exclusion may be considered if a clear embolic source is recognized [8]. Statins can be used as secondary prevention to improve the lipid profile in patients with CES; meanwhile, routine usage of anticoagulation is not recommended except if the patient had another indication for anticoagulation like atrial fibrillation or mechanical prosthetic valve [9-10].

Scolari et al. followed up 354 patients diagnosed with AERD from diagnosis until dialysis or death. They reported a 29% mortality rate, and 32.7% of the patients required dialysis [11]. CES had high mortality and morbidity as reported by Scolari et al. and the patients should be closely followed up to avoid complications and improve the quality of life. 

## Conclusions

Cholesterol embolization syndrome is a rare condition characterized by arterio-arterial embolization of cholesterol crystal or atheroma to small or medium arteries resulting in tissue ischemia or end-organ damage. It usually occurs after a cardiac or vascular intervention. We present a case of a 90-year-old Caucasian female who did not undergo any intervention and was diagnosed with CES resulted in AERD. This case highlights the importance of including CES and AERD as a cause of renal failure even if the patient did not have any recent cardiac or vascular intervention.
